# Role of Radiology in the Diagnosis and Treatment of Breast Cancer in Women: A Comprehensive Review

**DOI:** 10.7759/cureus.70097

**Published:** 2024-09-24

**Authors:** Muhammad Arslan, Muhammad Asim, Hina Sattar, Anita Khan, Farsina Thoppil Ali, Muneeza Zehra, Keerthi Talluri

**Affiliations:** 1 Medicine, Leeds Teaching Hospitals, NHS Trust, Leeds, GBR; 2 Emergency Medicine, Royal Free Hospital, London, GBR; 3 Medicine, Dow University of Health Sciences, Karachi, PAK; 4 Medicine, Khyber Girls Medical College, Peshawar, PAK; 5 Medicine, Kerala University of Health Science, Kerala, IND; 6 Internal Medicine, Karachi Medical and Dental College, Karachi, PAK; 7 General Medicine, GSL (Ganni Subba Lakshmi garu) Medical College, Rajahmundry, IND

**Keywords:** artificial intelligence, breast cancer, deep learning, image-guided therapy, mammography, mri, radiology, radiomics, theranostics, ultrasound imaging

## Abstract

Breast cancer remains a leading cause of morbidity and mortality among women worldwide. Early detection and precise diagnosis are critical for effective treatment and improved patient outcomes. This review explores the evolving role of radiology in the diagnosis and treatment of breast cancer, highlighting advancements in imaging technologies and the integration of artificial intelligence (AI). Traditional imaging modalities such as mammography, ultrasound, and magnetic resonance imaging have been the cornerstone of breast cancer diagnostics, with each modality offering unique advantages. The advent of radiomics, which involves extracting quantitative data from medical images, has further augmented the diagnostic capabilities of these modalities. AI, particularly deep learning algorithms, has shown potential in improving diagnostic accuracy and reducing observer variability across imaging modalities. AI-driven tools are increasingly being integrated into clinical workflows to assist in image interpretation, lesion classification, and treatment planning. Additionally, radiology plays a crucial role in guiding treatment decisions, particularly in the context of image-guided radiotherapy and monitoring response to neoadjuvant chemotherapy. The review also discusses the emerging field of theranostics, where diagnostic imaging is combined with therapeutic interventions to provide personalized cancer care. Despite these advancements, challenges such as the need for large annotated datasets and the integration of AI into clinical practice remain. The review concludes that while the role of radiology in breast cancer management is rapidly evolving, further research is required to fully realize the potential of these technologies in improving patient outcomes.

## Introduction and background

Breast cancer continues to be one of the critical health concerns worldwide and holds the record as the prevalent form of cancer among women and a major cause of cancer-related deaths. Technological advances in radiological imaging that have been accompanied by artificial intelligence (AI) in recent years have played major steps in improving the specificity and accuracy in diagnosis and management of breast cancer [1]. The current more common imaging techniques for screening of breast cancer have been mammography, ultrasound (US), and magnetic resonance imaging (MRI). Mammography, specifically digital mammography (DM), is the most popular screening method and has played a critical role in the early detection of breast cancers. However, its sensitivity can be relatively low in women with dense breast tissue mass since some lesions can be masked by other overlapping tissues [2]. Another essential tool is US because it is applied together with mammography in cases in which mammographic changes are ambiguous or in patients with large and dense breasts. In the last few years, better capabilities of characterization of breast lesions have been added to US because of high-frequency transducers and Doppler imaging. Also, the adoption of AI and deep learning in US imaging has been embraced due to their ability to enhance diagnosis and decrease observer variation [3,4].

Moreover, the specialization of radiological diagnostic and treatment procedures is experiencing the development of a contemporary innovative field identified as theranostics. Theranostics can be defined as the use of a diagnostic tool that is used to deliver a therapeutic agent when such tool is needed for diagnosis or when diagnosis is to be used for the delivery of the therapeutic agent; the concept of theranostics is especially valuable in cancer treatments, hence the use of the term “nanotheranostics.” These approaches employ nanoparticles that can be visualized by imaging technologies such as MRI or positron emission tomography/computed tomography (PET/CT) and that can also deliver therapeutic agents to the locus of the tumor and increase the effectiveness of treatment while at the same time minimizing toxicity to the rest of the body [5].

Furthermore, these technologies are promising, but the effectiveness of applied interventions should be investigated in various samples and settings. Also, the effects of these approaches on patients’ outcomes over time should be further explored. Thus, the contribution of radiology in the management of breast cancer is rapidly growing due to challenges in imaging technology, AI, and radiomics. In this paper, the authors sought to give an account of these advances so as to show that these tools can improve not only the diagnostic accuracy but also the precision of treatment, hence improving the outcomes of the patients.

## Review

Use of radiomics in detecting breast cancer

**Figure 1:**
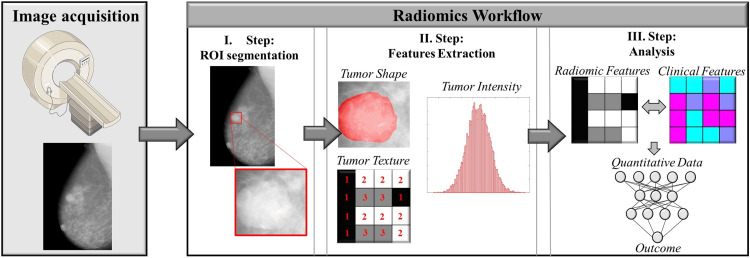
The image displays the radiomics workflow. I Step: ROIs are segmented automatically as the first step. In this instance, an ROI has been identified and outlined around a worrisome lesion. II Step: radiomic characteristics are derived from this ROI during the second stage. The features include the form of the tumor, as well as statistical characteristics generated from the image intensity histogram (referred to as first-order statistics features) and additional statistical characteristics known as “texture features” (referred to as second-order statistics features). III Step: in the third stage, radiomics characteristics are adjusted to eliminate redundancy. Significant quantitative characteristics are further examined using statistical techniques or machine learning algorithms to get a clinically understandable result. Reproduced with permission from Conti et al. [17] ROIs, region of interest

Radiomics, originally developed for imaging cancers of the head and neck and lungs, has only just been used in breast imaging [6,7]. Figure 1 shows a typical radiomics workflow. The need to improve the accuracy of traditional radiological procedures such as mammography, tomosynthesis, and MRI motivates one possible use of radiomics [8]. Radiomics' capacity to distinguish between normal breast tissue and malignant growths, as well as between benign breast lesions, has been the subject of several studies. The results of these studies suggest that breast imaging diagnosis might be improved by including radiomics in the conventional radiological procedure. One useful criterion for differentiating between benign and malignant tumors was found to be entropy, as evaluated in an MRI-based radiomics study. More vascular activity and heterogeneity inside the tumor was indicated by greater entropy values in malignant lesions [9]. Using a set of quantitative factors derived from MR images, another study used dynamic contrast-enhanced MRI to differentiate between benign breast lesions and luminal breast tumors [10]. Using 38 retrieved characteristics, an analysis was performed on 508 lesion DCE-MRI images. The findings showed that the area under the curve (AUC) was 0.797 when focusing just on the maximum linear size. The AUC became better at 0.846 and 0.848, respectively, when size features were included or excluded from the feature selection processes [11]. AUC values between 0.842 and 0.851 show that several radiomics classifiers effectively differentiate between benign and malignant tumors. The results obtained are lower than those attained by experienced breast radiologists (AUC of 0.959); hence, further evaluation is necessary to define the utility of radiomics in distinguishing between benign and malignant tumors [8]. Bickelhaupt et al. conducted a multicentric prospective analysis using unenhanced MRI sequences to assess a radiomics model of suspicious breast lesions derived from breast tissue and reported positive outcomes. Using radiological X-ray methods, researchers in a prospective, multicenter study digitally scanned women with dense breasts utilizing breast tomosynthesis, employing a radiomics approach. The purpose of this pioneering research was to analyze radiomics in this particular medical context [12]. This research study included 20 individuals who initially tested negative for breast cancer by standard mammography but were subsequently revealed to have the disease by digital breast tomosynthesis (DBT) [13]. The malignancy was then confirmed by histology. In addition, 20 individuals who were tested for breast cancer using DBT but tested negative were included as a control group. Both the age and breast density of these individuals were comparable. Three parameters (skewness, entropy, and 90 percentile) out of 104 radiomics characteristics examined demonstrated statistically significant variations between the two sets of data [14]. Radiomics analysis of DBT images may be helpful in cancer detection, according to the initial encouraging results. An AI system that used deep learning and other machine learning algorithms was able to achieve cancer diagnostic accuracy comparable to that of a human breast radiologist working in a retrospective setting, according to reports [15]. The nine datasets included in this study were from seven different countries and were used for different types of research in the past. Multiple readers, cases, and vendors were included in the datasets. There was a grand total of 2,652 examinations (including 653 cases of cancer) together with the expert opinions of 101 radiologists (a total of 28,296 distinct interpretations), all of which were DM radiological tests that were double-checked by histological investigation or follow-up. After running the tests, the AI system achieved an AUC of 0.840 (95% CI = 0.820 to 0.860). When comparing, the radiologists had an average AUC of 0.814 (95% CI = 0.787 to 0.841), a difference ranging from 95% CI = -0.003 to 0.055. A large percentage of radiologists outperformed the AI model because the AI system attained an AUC higher than 61.4% of the radiologists. The study's authors agreed that more research is necessary to determine the system's efficacy and the consequences of using it in a screening environment. Nevertheless, current results are promising and might greatly enhance breast diagnostic imaging and screening; nevertheless, more research is needed to validate its generalizability. AI algorithms were recently the subject of a feasibility study to ascertain whether or not they might automatically identify normal DM examinations, therefore reducing the burden of reading breast cancer screening findings [16]. The authors of this research speculated that AI may be used to automatically filter out breast cancer screenings that had a low likelihood of malignancy, thereby reducing the burden on human readers. On top of that, radiologists may still be able to identify breast cancer even after excluding screening tests with the lowest cancer probability. Further research is needed to address this issue, particularly with the practicality of using this method in actual screening settings (Figure 1).

Diagnosis of breast cancer

Ultrasound

Similar to DM and DBT, deep learning was first used in the US to classify tumors as benign or malignant [18]. In 2017, a modified version of the GoogLeNet Convolutional Neural Network (CNN) was trained using US pictures of problematic lesions' regions of interest (ROIs) [19]. AUC for this model was 0.960, indicating outstanding performance. With the same dataset, a classic computer-aided detection (CAD) system that used features created by hand achieved an AUC of 0.90. A later step included applying deep learning to the whole US image for direct categorization [20-22]. Results showed AUC values between 0.84 and 0.95, which is similar to ROI categorization. However, American datasets tend to be smaller than disease management sets, mostly due to the fact that screening is not as common in the US. A well-known and successful method for improving performance when dealing with restricted datasets is data augmentation, which is also known as transfer learning. Data augmentation is the process of adding new data to an existing dataset by manipulating it in various ways, such as rotating, flipping, and shifting. The majority of the studies discussed here really make use of some kind of data augmentation. When deciding which kind of augmentation to use in the US, it is crucial to proceed with care. According to Byra et al. [18], performing a longitudinal shift or rotation on the images might change the recognized features of breast lumps, which may result in less accurate classifications. The reason for this is that lesions with a horizontal orientation (wider than tall) are less likely to be malignant than those with a vertical orientation (taller than broad).

The Breast Imaging-Reporting and Data System (BI-RADS) is used by radiologists to classify suspicious lesions, instead of classifying them as benign or malignant [23]. From 1 (no suspicious findings) to 5 (greater than 95% risk of cancer), the BI-RADS categorization system spans a wide range of results. In order to determine the best way to treat a patient, this categorization method takes into account the probability of cancer. Lesions classified as BI-RADS 3 are appropriate for the watchful waiting period since the probability of cancer is less than 2%. In contrast, a biopsy is often necessary for lesions that are BI-RADS 4 or 5. Mistakes in lesion categorization and observer bias continue to be problems with the breast US classification approach. Consequently, CADs are created to assist radiologists in selecting the correct BI-RADS category. These categories are based on a variety of sources, including pathological classification and radiologists' subjective assessments [24,25]. However, I have my doubts about the second approach: There is already the possibility of inter-observer variability and structural probability of malignancy misclassification in the training data of these CADs. Following the logic of DM, it is possible to combine detection and classification into a single network [26].

Researchers conducted a comprehensive experimental study to find out if famous CNN-based deep learning architectures do the best when it comes to US image recognition and classification. When it came to categorizing full-image images, DenseNet achieved an accuracy of 85%, and for pre-defined ROIs, it reached an accuracy of 87.5%. The Faster R-CNN model was used by Shin et al. to construct a separate network for the two tasks using semi-supervised learning [27]. Training a network using both sparsely and highly annotated photographs may considerably improve performance, according to the findings, much more so than training with simply a comparable number of highly annotated images.

In order to precisely ascertain the size and extent of lesions, deep learning may be used for lesion segmentation in both 3D automated breast US scans and handheld US images [28]. For the purpose of segmenting images of biological objects, the U-net is a popular segmentation network. Since half of the segmentation references mentioned earlier employ a U-net, or a modified version of it, there is evidence that the U-net is useful in breast cancer imaging. But, newly developed segmentation networks in fields other than biomedical imaging, such as the YOLO network, also show great potential in this field. The capacity of these networks to detect anomalies, partition them into independent components, and ascertain whether they are benign or malignant is bringing them closer to use in clinical practice.

**Figure 2:**
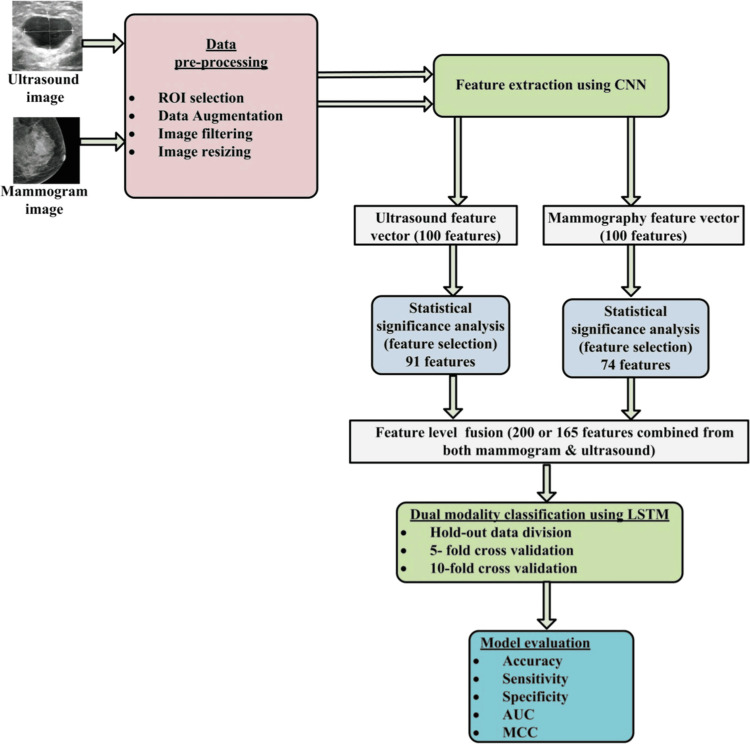
Hybrid CNN-LSTM model for bimodal BC classification. Reproduced with permission from Atrey et al. [29] BC, breast cancer; CNN, Convolutional Neural Network; LSTM, long short-term memory; ROI, region of interest

Conventional approaches to diagnose breast cancer are prone to human mistakes, have lower accuracy, and are time-consuming. A CAD system may address the aforementioned shortcomings and assist radiologists in making precise decisions. Nevertheless, previous research using just one kind of imaging technique has shown restricted clinical use owing to its diminished diagnostic precision and dependability in comparison to a multimodal system. An integrated model, consisting of a CNN and long short-term memory (LSTM), is used to enhance the early detection of breast cancer by incorporating pictures from mammography and US modalities. This study uses a novel dataset comprising of 43 mammography pictures and 43 US images obtained from 31 individuals in real-time. In addition, each group has 25 benign photos and 18 cancerous ones. The total number of photos has been augmented to 1,032, including 516 images for each modality, by the use of several data augmentation methods. With an AUC of 0.99 and a classification accuracy of 99.35%, the suggested bimodal CAD method is a formidable contender. Contrast this with the classification accuracies of 98.84% when using US and 97.16% when using mammography, produced by conventional unimodal CAD systems. The suggested bimodal CAD method, which combines mammography and US, demonstrates superior performance compared to standard unimodal CAD systems. The bimodal CAD method has the ability to prevent needless biopsies and promote its use in clinical settings [29]. Figure 2 illustrates the implementation of a semi-automated bimodal CAD system for breast cancer categorization, which uses the CNN-LSTM model [29].

Magnetic Resonance Imaging

Like DM, US, DBT, and DL are primarily used in the MRI area for identifying, classifying, and segmenting breast lesions. The dimensionality of these modalities, however, is the main differentiator from MRI. In contrast to MRI's three-dimensional (3D) scans, those produced by DM, DBT, and US only show two dimensions. The complexity is further increased to a four-dimensional (4D) level by MRI sequences that document changes across time, such as when contrast agents are inserted or removed from the body (DCE-MRI). In medical applications, applying deep learning models trained on two-dimensional (2D) images to 3D or 4D MRI scans might be challenging.

A number of approaches have been used to deal with these problems. An often-used technique is flattening the 3D images so that standard 2D deep learning models may be employed. The maximum intensity projection (MIP) method or 3D MR image segmentation into 2D slices are two possible approaches. Having said that, a number of traditional deep learning models are tailored to color images, particularly those with RGB (red, green, and blue) channels. Therefore, these models use a 3D image with the three color channels making up the third dimension as input. MRI scans only use one color channel since they are constructed of greyscale images. This means that three MIPs or slices may be used as one input picture. This paves the way for creating an MRI input picture with three successive slices that is partly 3D or for combining several post-contrast slices or MIPs into one input image. Alternatively, you may use DenseNet or another model specifically built for 3D data or may alter existing 2D deep learning models to include 3D data using real 3D MRI scans. When compared to AI algorithms that can use the actual 3D or 4D input of breast MRI, strategies that first apply dimension reduction are anticipated to have lower performance. However, as of right now, there is zero proof of this improvement.

In reality, all of these methods have been used in the MRI lesion categorization research. Several teams fed in 2D ROI slices as their starting point [30,31]. They were able to get AUC values between 0.908 and 0.991 [32]. Researchers demonstrated AUC values ranging from 0.84 to 0.92 in a study that used the three RGB channels and the inclusion of many post-contrast slices, namely slices [33,34]. In conclusion, a number of studies found AUC values of 0.859 and 0.852 respectively, using actual 3D MRI images [35]. It may seem that the AUCs are becoming smaller as we go from using simple 2D approaches to more complex 3D ones, as they are consistent across research. Because each of the aforementioned research used a distinct dataset, it is not possible to directly compare these numbers. However, studies that used other approaches have compared their results. While AI models' sensitivity was about the same or slightly lower than that of radiologists, their specificity was much higher.

Deep learning is not only employed for lesion classification; MRI is also used to categorize metastases to the axillary lymph nodes [36,37]. Instead of using biopsy results as a benchmark, two of these studies employed PET. Despite its status as the gold standard, the researchers noted that artifacts such as needle marks or biopsy clips might be present in a biopsy, leading the deep learning algorithm to incorrectly identify the sample as cancerous. They outperformed the radiologists with accuracies of 84.8% and 88.5%, respectively, by using PET as the reference standard. PET is often used in clinical practice to diagnose aberrant lymph nodes.

Deep learning has also shown great strides in lesion segmentation and detection, much like other modalities. When it came to creating a model that used deep learning to identify lesions in MRI data, Maicas et al. were among the first [38]. They used deep Q-networks to train a network to gradually reduce the size of the box to contain the lesion if a lesion is found within the initial large bounding box. Their deep Q-network achieved results that were on par with those of conventional exhaustive search methods, but it used four times as little computational power. To identify lesions in 4D MR data, Ayatollahi et al. employed a unique approach by training RetinaNet, a specialist network for detecting small objects in images [39]. The sensitivity rate they achieved was 95%, with just four false positives per breast.

It is worth mentioning that a breast mask was originally used in 50% of the MRI detection and segmentation procedures stated earlier. You might think of these breast masks as complete breast enclosures. Using them in conjunction with an MRI scan selectively lets through breast-related pixels while setting all other pixels to zero. There are two purposes for these masks. To start, they may tell the detection and segmentation deep learning algorithms to zero in on the correct areas. The masks may also be used to determine the mammographic density, a crucial marker for a higher likelihood of breast cancer. Thus, the fact that deep learning is involved in complete breast segmentation should come as no surprise [40]. Results showed that segmenting lesions was more difficult than the whole breast (Dice scores ranged from 96 to 99 in the studies). The density of mammographic images is defined as the percentage of breast fibroglandular tissue (FGT). The breast mask and segmented FGT may be used to conduct the computation. For FGT sections [41,42], deep learning is used to segment the FGT with the goal of autonomously determining mammographic density. For example, prior atlas-based approaches were compared with Dalmıs et al.'s deep learning approach that used a U-net [43]. By comparison, the Dice similarity coefficient for the earlier atlas-based approaches was 0.671, whereas their deep learning method had a value of 0.850. In a different research, neoadjuvant chemotherapy (NAC) response prediction is another area that might benefit from deep learning. Some studies simply employ DCE-MRI scans captured before NAC treatment begins, while others use a mix of scans taken before and during the start of NAC therapy [44].

Pathological complete response (pCR) after NAC in HER2-positive (HER2+) breast cancer patients was the goal of a retrospective multicenter research conducted by Braman et al., which included 5 institutions [45]. For two different testing datasets, the researchers were able to reach AUC values of 0.77 and 0.85, showing much improved performance compared to traditional pCR NAC prediction methods. This shows how deep learning can be used in this kind of context. In MRI, generative adversarial networks (GANs) have shown their worth. Because different brands of MR scanners produce different noise distributions and intensity values, models trained on one set of scans may not be able to generalize to the other, which may result in a decline in performance. To standardize the MR images acquired from different vendors, Modanwal et al. used a GAN [46]. Using MRI data acquired from two different suppliers, the GAN was trained to understand the bidirectional association. In their demonstration of Deep Learning's potential for MRI normalization, the authors achieved bidirectional Dice similarity coefficients of 0.98. When it came to the issue of fat suppression in DCE-MRI pictures, Mori et al. looked at a particular sort of GAN called pix2pix GAN [47]. Most of the time, the existing fat-suppression method shows signs of non-uniformity due to the MRI scanner's uneven magnetic field. Researchers developed a model using the pix2pix approach to generate bogus fat-saturated T1-weighted images from non-contrast-enhanced T1-weighted data. A pair of breast radiologists evaluated the synthetic pictures on a scale from 1 (very good) to 5 (very bad). When compared to pre-existing fat-saturated T1-weighted pictures, the synthetic images scored 3 out of 5. The average score of 3.12 for the synthetic pictures further shows how deep learning might impact breast cancer imaging.

**Figure 3:**
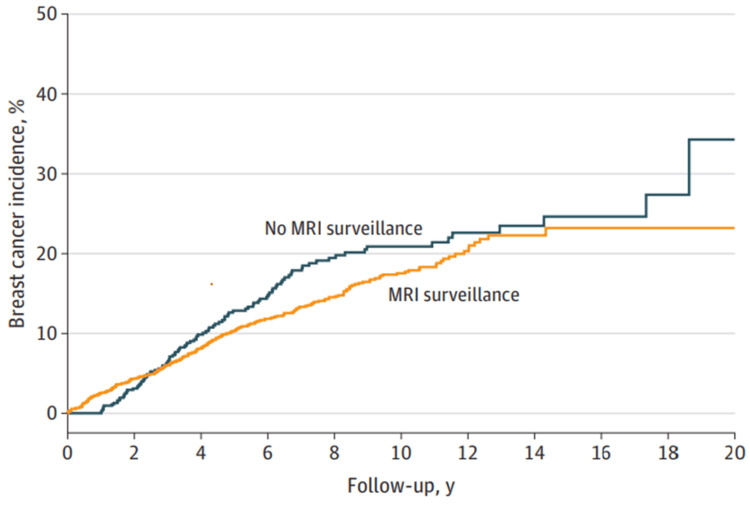
Risk of breast cancer related death in women carrying a BRCA1 or BRCA2 variant, broken down by MRI surveillance status over a 20-year period. Reproduced under the terms of the CC-BY License from Lubinski et al. [48]

In order to monitor the elevated risk of breast cancer in women with pathogenic *BRCA1* or *BRCA2* gene mutations, MRI is used. MRI surveillance has the potential to reduce the size of breast cancers, although the effect on the risk of death is not yet known. Lubinski et al. evaluated how well MRI monitoring detects breast cancer in women with *BRCA1 *or *BRCA2 *mutations compared to women who did not undergo the monitoring program. A total of 2,488 women with mutations in the *BRCA1* or *BRCA2* genes were included in the research. Their ages ranged from 30 to 69 years, and the mean age at study enrollment was 41.2 years. Out of the total number of women surveyed, 1,756 (or 70.6% of the total) had undergone a screening MRI, whereas 732 (or 29.4%) had not. Following an average follow-up of 9.2 years, 35 (1.4% of the total) women passed away and 344 (13.8%) had breast cancer. For women carrying *BRCA1* sequence variants, the breast cancer mortality rate associated with MRI surveillance programs was 0.20 (95% CI, 0.10-0.43; P < 0.001), and for those carrying *BRCA2* sequence variations, it was 0.87 (95% CI, 0.10-17.25; P = 0.93) (Figure 3) [48]. ​​

Nuclear Medicine Imaging

When it comes to assessing early-stage breast cancer, DM, BDT, MRI, and US are superior to nuclear medicine imaging modalities such as PET and scintigraphy. However, there are additional advantages of using nuclear medicine technologies for finding and classifying lymph nodes in the armpit region and for assessing the disease's prevalence in faraway places [49]. It should come as no surprise, therefore, that deep learning finds some limited use in this area of imaging as well. One possible way to accurately measure the tumor's extent is to quantify its metabolic tumor volume (MTV) throughout the body using PET/CT. Because in clinical practice acquiring the MTV requires identifying all tumors, using a deep learning model for this procedure drastically reduces the need for human effort. Weber et al. tested a CNN's ability to identify and localize breast cancer patients' lesions in their whole-body PET/CT scans [50]. The CNN had already been trained to compute the MTV for patients with lymphoma and lung cancer. This neural network's adaptability to various scenarios was shown by the statistically strong relationship between the MTVs generated by deep learning and those that were split manually. However, the deep learning model did not succeed in correctly recognizing all lesions. Overall, its sensitivity for detecting all lesions was just 39%, but it did a good job of identifying 92% of PERCIST quantifiable lesions.

Bone metastases are more often caused by prostate and breast cancers. In order to reliably identify breast cancer metastases in whole-body scintigraphy scans, Papandrianos et al. used a robust CNN [51]. They had a 92.5% success rate by labeling all of the body scans as benign or cancerous. Furthermore, they evaluated DenseNet against preexisting CNN designs and found that it obtained the maximum achievable accuracy of 95%. Furthermore, deep learning and nuclear medicine technologies are used in some breast cancer imaging techniques to enhance the performance of procedures. A 3D CNN model was created by Li et al. to assist doctors in identifying metastases to the lymph nodes in the axilla on PET/CT scans [52]. On average, doctors' sensitivity increased by 7.8 percentage points when they used their network, while their specificity stayed at 99.0%. However, both doctors were able to outperform the deep learning model on its own. For patients with advanced breast cancer, Choi et al. showed that a deep learning network trained on PET/MRI images may predict pathological reactions to NAC [53]. The AUC values of the CNN based on Alexnet were superior when compared to conventional response indicators to NAC, namely MTV and maximum standardized uptake value. Notably, their dataset was rather limited, consisting of just six respondents and 50 non-responders.

Novel therapies and current treatment options for breast cancer subtypes

Multiple imaging modalities, including breast examinations, mammography, US, MRI, and others, may identify breast cancer. As mentioned earlier, these techniques work well for finding breast tumors and other abnormalities. A mass, a region of tiny calcium deposits, an area that would be worrisome on US, or an area that would be emphasized by gadolinium on MRI might be detected with the use of these imaging modalities. Rapid and intense treatment is required to eradicate the tumor and stop the disease's advancement at the discovery of breast cancer using any of the aforementioned diagnostic methods. The discipline faces a substantial difficulty due to the variability in the nature and response to treatment of breast cancer [54]. By searching for indicators such as hormone receptors (HRs), elevated HER2 protein levels, and/or more HER2 gene copies, it is possible to ascertain the optimal treatment plan for a certain subtype of breast cancer [55]. Breast cancer is classified into five main molecular subgroups based on the overexpression of certain genes.

Luminal B tumors that show high levels of the protein Ki-67 and are positive for HRs (estrogen receptor [ER] and/or progesterone receptor) are typified by luminal B breast cancer. For the HER2 protein, these tumors might be HER2-negative (HER2-) or HER2+. When compared to luminal A breast cancer, the luminal B subtype is often more aggressive and has a greater cell proliferation rate. Consequently, luminal B breast cancer has a somewhat worse prognosis [56].

HER2 overexpression is not present in triple-negative or basal-like breast cancer, and the tumors do not have any estrogen or progesterone receptors. There is an increased risk of developing this malignancy in women who have *BRCA1* gene mutations [57].

When breast cancer tumors test positive for HER2 but negative for estrogen and progesterone receptors, we say that the tumors are HER2-enriched. This subtype is distinguished from luminal tumors by its tendency to proliferate rapidly [58]. Enhertu (fam-trastuzumab-deruxtecan-nxki), Tykerb (lapatinib), Herceptin (trastuzumab), Perjeta (pertuzumab), and others are medications that target the HER2 protein [59], may be a successful treatment for patients.

Cancers that mimic normal breast tissue have a lower level of the Ki-67 protein, are HER2+, and are HR+ estrogen- and/or progesterone-receptor positive). Luminal A cancer and this sort of cancer are quite similar. The prognosis for luminal A illness is somewhat better than that for normal-like breast cancer.

The prodrug tamoxifen (brand name Nolvadex) inhibits estrogen absorption by the ER [60], making it a partial agonist. According to certain studies, tamoxifen may reduce the risk of ER+ breast cancer recurrence by half [61]. Tamoxifen has been associated with an increased risk of endometrial cancer, blood clots, and stroke, among other well-documented side effects, as are many other anti-cancer medicines [62]. In postmenopausal women, aromatase inhibitors reduce estrogen synthesis by blocking the conversion of androgens to estrogens. A total of three distinct aromatase inhibitor implementations have been developed. The first generation of aromatase inhibitors, such as aminoglutethimide, as well as the second generation, including fadrozole and vorozole, show reduced selectivity in inhibiting aromatase and affecting cortisol and aldosterone synthesis. Furthermore, their usefulness in clinical settings is limited, and they are not very well-tolerated [63]. In contrast, third-generation aromatase inhibitors such as exemestane (Aromasin), anastrozole (Arimidex), and letrozole (Femara) are very selective to the aromatase enzyme and often have little side effects. As a result, they have shown better response rates and delayed disease progression than tamoxifen in the treatment of HR+ metastatic breast cancer in postmenopausal women. In comparison to tamoxifen, AIs have shown a number of advantages, including a slower rate of reduced occurrence of breast cancer on the other side, local and metastatic recurrence, and progressive improvement while the patient is healthy [64].

Goserelin and leuprolide are examples of luteinizing hormone-releasing hormone (LH-RH) analogs that decrease ovarian hormone production [65]. Gonadotrophin secretion is inhibited by LH-RH agonists because they trigger pituitary desensitization and receptor downregulation [66]. The suppression of ovarian steroid synthesis and release is unrelated to the direct anticancer impact of LH-RH on malignant tissue [67]. Patients with breast cancer who have not shown improvement after previous hormonal treatments may choose to consider fulvestrant, a selective ER degrader. The first selective ER downregulator that can be used therapeutically is this one. Without inducing any stimulating effects, this anti-estrogen molecule targets and destroys ER alpha (α). In addition, it works in tamoxifen-resistant breast cancer models [68]. Fulvestrant, the first selective ER downregulator to enter clinical practice, provides a solid basis for endocrine management of breast cancer patients undergoing combination treatment with new targeted medications. In ER+ breast cancer cell lines, fulvestrant has been shown to decrease ERα expression in preclinical studies. This reduction in activity not only hinders the activity of ER-responsive genes but it also happens independently of the ERα gene transcripts [69]. Fulvestrant may prevent estradiol from acting on the G-protein coupled ER (GPER), a distinct kind of ER from the two primary ERs (ERα and ERβ), in a way that is not genetic. It is hypothesized that GPER, which is present in 50-60% of breast cancer cases, is linked to the development of tamoxifen resistance in individuals with ERα+ breast cancer. These mechanisms restrict the ability of ER+ breast cancer cells to multiply. A number of cell lines that are resistant to tamoxifen have also shown that fulvestrant is effective [70]. A xenograft model developed from an ER+ breast cancer patient validated fulvestrant's ability to limit tumor development. Since it outperforms tamoxifen and estrogen withdrawal, we may conclude that it is superior [71]. Because endocrine medicines have several mechanisms of action, they are often used in combination to increase the anticancer efficacy of these drugs. Having said that, there are discoveries that go against one another. Patients whose tumors are very receptive to endocrine treatment or who have advanced breast cancer and have not had prior endocrine therapy may benefit most from combination endocrine therapy [72,73]. As will become clear in the subsequent explanation, a number of other indicators have been recognized as potential targets for the therapy of breast cancer.

Circulating Cyclin-Dependent Kinases 4/6 Route

By assembling complexes with cyclin D proteins, which regulate cellular processes during the G1 phase of cell cycle, CDK4/6 plays an essential function in enhancing cell proliferation. If this regulation of the cell cycle can be better understood, it could lead to new cancer therapies [74]. The efficacy and safety of drugs that inhibit CDK4/6 activity in the treatment of breast cancer are the subject of many active studies [75]. The development of flavopiridol, a pan-CDK inhibitor, came to a stop because of severe side effects and decreased effectiveness [76]. Researchers shifted their attention to creating extremely selective inhibitors, such as ribociclib, palbociclib (PD0332991), and abemaciclib [77,78]. As of now, palbociclib and ribociclib may be used to treat HR-positive, human epidermal growth factor receptor 2-negative, or metastatic breast cancer, according to the U.S. Food and Drug Administration (FDA). Combining palbociclib with either letrozole or fulvestrant significantly improved the clinical outcome, according to recent clinical trial results [79,80].

Phosphoinositide 3-Kinase Route

One of the most common signaling pathways activated in human cancer is the PI3K pathway, which stands for phosphatidylinositol 3-kinases. There is a family of enzymes that facilitate communication between oncogenes and different types of receptors involved in cellular activities. They play a crucial role in the transmission of signals [81]. An important mechanism in cancer formation and progression is the PI3K/AKT/mTOR pathway, which is also called the phosphatidylinositol-3-kinase/AKT/mammalian target of rapamycin pathway [78]. By focusing on adenosine triphosphate (ATP), pan-PI3K inhibitors bind to different PI3K isoforms selectively and competitively. For advanced breast cancer that is HR+/HER−, PI3K inhibitors as an additional course of therapy once aromatase inhibitors have been exhausted. Patients with a range of solid malignancies, including breast cancer, are now participating in clinical studies for the oral pan-class I PI3K inhibitor buparlisib [82,83]. Patients with HR+, HER2-advanced breast cancer who have seen progression during or after mTOR inhibitor treatment should not be treated with buparlisib due to the toxicities associated with the drug, according to a new study. Patients with PIK3CA mutations still have hope for a therapy plan that combines PI3K inhibitors with endocrine treatment, as the medication's success suggests [84].

Pictilisib is an additional pan-PI3K inhibitors that shows decreased inhibition of the p110β and -γ isoforms but equivalent inhibition of the p110α and -δ PI3K isoforms [85]. Pictilisib was ultimately determined to be safe for patients in a phase I dose-escalation clinical trial that included 60 patients with advanced solid malignancies. Hyperglycemia, dermatitis, and pneumonitis were among the serious adverse effects that were reported with it [86]. Antineoplastic promise exists for the small chemical pilaralisib, also known as XL147, which may be given orally [87]. XL147 inhibits tumor cell proliferation in sensitive tumors by reversibly binding to class 1 PI3Ks, which it particularly targets. It is common for the PI3K signaling pathway to be activated during tumor formation. The pharmacokinetics, safety, and effectiveness of pilaralisib and voxtalisib, two PI3K inhibitors and mammalian targets of rapamycin, in conjunction with letrozole were examined in a Phase I/II dose-escalation trial. Patients whose breast cancer was HR+, HER2-, non-steroidal AI-refractory, recurrent, or metastasized were the primary subjects of the research. The glucose levels of patients treated with pilaralisib were greater than those of patients treated with voxtalisib. The results demonstrated that patients with HR+, HER2-metastasizing, and endocrine-therapy-resistant breast cancer had a moderate efficacy and a good safety profile when given pilaralisib or voxtalisib in conjunction with letrozole [88].

Aiming HER2+ Breast Cancer Cure

Drug resistance and an increased risk of metastasis are hallmarks of HER2+ breast cancer, often referred to as HER2+ breast cancer. The prognosis of individuals with HER2+ breast cancer has been significantly improved by targeted therapy medications. Drug resistance and serious side effects, however, have limited these treatments' clinical use. Several avenues are currently being explored to address pharmaceutical resistance and provide a more efficient treatment. The HER2 oncogene, which goes by many names (HER2/neu, c-erbB-2, and just HER2), is located on chromosome 17, as noted before [59,89]. Its principal function is to code for a receptor tyrosine kinase that can cross cell membranes [90]. Many cellular activities depend on the transmission of signals between cells; these include cell motility, metabolism, differentiation, and proliferation. These processes rely heavily on tyrosine kinase receptors. Receptors consist of an extracellular domain that binds to ligands, a transmembrane helix, and an intracellular domain that acts as a tyrosine kinase. Additionally, receptors have a juxtamembrane area and a carboxy terminal tail [86]. The competitive blocking of tyrosine phosphorylation and tyrosine kinase enzyme activity is the mechanism by which tyrosine kinase inhibitors (TKIs) affect several cellular functions [91]. The irreversible suppression of HER1/HER2/HER4 is the hallmark of the TKI neratinib, which was created in the United States by Puma Biotechnology, Inc. According to research, individuals with HER2+ breast cancer who have had adjuvant therapy with trastuzumab have a significantly higher two-year invasive disease-free survival rate [92]. On February 25, 2020, the FDA approved neratinib to be used in combination with capecitabine. This clearance is for the exclusive use of treating patients with metastatic HER2+ breast cancer who have already undergone two or more anti-HER2 based therapy regimens. TKIs such as lapatinib work by blocking ATP-binding sites within cells in a competitive manner. According to a study, it effectively and reversibly inhibits HER1 and HER2 phosphorylation [93]. In a phase III clinical trial, patients with HER2 metastatic breast cancer who were given lapatinib in addition to the anti-neoplastic medicine paclitaxel had a far better chance of survival [94]. Another pharmacological component, tucatinib, showed more HER2 selectivity than earlier TKIs in a phase I study of patients with advanced disease. Diarrhea was also less common among patients.

 There are significant epidemiological, clinical, and prognostic differences between HER2+ breast cancer and HER2- cancers; HER2+ breast cancer is a subtype that is far more aggressive. Additionally, it has a low reactivity to standard chemotherapy, leading to unsatisfactory results [95]. As a recognized sign of invasive disease that is prone to substantial metastases, treatment resistance, and quick dissemination, HER2 has been evaluated in around 30% of breast cancer patients [96]. The past 20 years have seen tremendous advancement in the creation of drugs for HER2+ breast cancer control, mostly in the form of targeted therapy dependent on HER2 expression level [97]. The HER2 ectodomain is the site of particular targeting for the humanized monoclonal antibody trastuzumab, better known as Herceptin. Patients with breast cancer who have an overexpression of HER2 have demonstrated improvement after using it. Trastuzumab efficiently blocked the basal and triggered HER2 cleavage, which led to the production of phosphorylated p95 [98]. Although both pertuzumab and trastuzumab bind to the HER2 dimerization domain, pertuzumab does so at a different epitope. This suppresses cell proliferation by preventing HER2 receptors from interacting with other HER2 family receptors [99,100]. One reason HER2-directed monoclonal antibodies are so successful against cancers is that they may directly target the extracellular domain of HER2.

The anticancer effects in preclinical animals were shown by the monoclonal antibody patritumab, which targets the HER3 receptor and prevents the development of HER2/HER3 heterodimers. Patients with advanced HER2+ breast cancer reported good efficacy and tolerability in the trial [101]. Determining the target trough level allowed us to construct the patritumab pharmacokinetic profile. We evaluated the drug's efficacy by looking at the total response rate and progression-free survival metrics.

Breast Cancer Treatment for Triple-Negative Patients

Only around 10-15% of breast cancer cases are categorized as triple-negative [102]. The lack of estrogen and progesterone receptors and low amounts of the HER2 protein are hallmarks of triple-negative breast cancer (TNBC) [103]. The invasiveness and proliferation of TNBC are much greater than those of other breast cancer subtypes, and the rate of tumor dissemination is also significantly faster. There is a worse prognosis and fewer treatment options for patients with TNBC [104]. For TNBC, conventional chemotherapy is still the go-to treatment. However, recurrence and metastatic rates are higher in TNBC tumors compared to non-TNBC tumors [105]. Carboplatin with a taxane drug, such docetaxel, improved the efficacy and toxicity profile of treatment for patients with advanced TNBC compared to docetaxel alone. Carboplatin yielded twice as many responses as docetaxel in individuals with germline *BRCA1/2*-mutated breast cancer. In order to pick the most effective treatment for initial chemotherapy, it is crucial to identify breast cancer patients who have a *BRCA1/2* mutation [106]. Because there are not many well-studied molecular targets for TNBC, therapy choices are limited when compared to those for other breast cancer subtypes. Finding new therapeutic targets and developing efficient tailored medications is, hence, an immediate need.

For the treatment of metastatic TNBC that has relapsed or is resistant to prior antibody-drug combinations, the FDA has approved sacituzumab govitecan. Trop-2 is a monoclonal antibody that specifically targets anti-trophoblast cell-surface antigen and was developed in conjunction with SN-38, an active irinotecan metabolite that inhibits topoisomerase I. The rationale for the approval was derived on the findings of the IMMU-132-01 phase I/II multicenter trial [107]. Merging the roles of an antibody and a topoisomerase inhibitor, Enhertu is a powerful medicine. Cancer cells expressing the HER2+ receptor are its intended targets. Adults whose HER2+ breast cancer has spread or is not amenable to surgery are the target population for Enhertu [108]. After receiving herceptin and taxane chemotherapy as neoadjuvant treatment for HER2+ metastatic breast cancer, patients may be treated with the FDA-approved drug Kadcyla, also known as T-DM1. Emtansine is connected to Herceptin in T-DM1, a targeted therapy that employs an antibody-drug conjugate [109].

Pembrolizumab, sold under the trade name Keytruda, is an immunotherapy drug that targets the programmed cell death 1 receptor. The antibody is highly selective and is a monoclonal IgG4-Ę. Patients with metastatic TNBC or recurrent TNBC that is not treatable by surgery were able to extend the time without disease progression when pembrolizumab was administered in conjunction with first chemotherapy [110]. For patients with TNBC, the use of pembrolizumab in combination with chemotherapy has been expedited by the FDA. Patients diagnosed with PD-L1+ TNBC may now begin therapy with atezolizumab and nab-paclitaxel, according to a new FDA authorization [111]. There is little doubt that breast cancer patients now have access to a wide range of imaging and therapy options. To improve the efficacy and individualization of breast cancer treatment, there is an increasing interest in combining diagnostic and therapeutic components into one system. What follows is an outline of the approaches that are now being studied in this domain.

Recent trends in breast cancer theranostics

Treatment for cancer has traditionally included locating tumor lesions with the use of an appropriate diagnostic imaging method and then deciding whether to use radiation, chemotherapy, or surgery. Unfortunately, there are some downsides to these treatments. For example, there is a possibility that the tumor may not be fully removed during surgery. Additionally, toxicities may occur that do not specifically target the disease. Abnormal blood vessel formation can lead to increased interstitial pressure and reduced blood flow, resulting in insufficient medication concentrations at the tumor site and limited drug penetration into the tumors [112]. In addition, studying the pharmacokinetics of chemotherapy by measuring drug concentration in plasma is a flawed approach [113,114], which is why conventional techniques for drug kinetic evaluations avoid it. The capacity of personalized medicine to tailor treatment to each patient's unique characteristics and needs has made it a hot topic in the past 20 years. The relatively new area of research known as theranostics was born out of this strategy to lessen the likelihood of adverse effects. An area of study known as "theranostics" focuses on developing more targeted approaches to treating cancer and other illnesses by combining diagnostic and therapeutic tools. This method enables the drug's efficacy to be tracked in real-time, which may enhance cancer treatment regimens. Because postponed treatment increases the chance of mortality, accurate identification is critical for rapid therapeutic action [115].

The use of a multi-functional nanotherapeutic system that can diagnose, provide targeted treatments, and monitor therapeutic response all at once is called theranostic nanotechnology or nanotheranostics [116]. Through the use of several imaging modalities, a single nanoparticle formulation may be followed when it penetrates cellular barriers in search of receptors that cancer cells have overexpressed. A fluorophore/contrast agent, therapeutic medications, and specific ligands are all connected to this formulation. At some point, the nanoparticle will release the medication into the tumor's surroundings in a controlled manner.

As a way to effectively manage breast cancer, nanotheranostics is now the subject of substantial investigation. Several imaging techniques may be used to follow the targeted accumulation and treatment of nanotheranostic formulations at the cancer site after they have been delivered. The versatility and biocompatibility of lipid-based carriers make them a popular choice. Examples of these include micelles and liposomes. Vitamin E-TPGS and Pluronic F127 block copolymer were used by Gregoriou et al. to produce theranostic micelles. In an effort to combat breast cancer, these micelles have shown promise as a specific delivery mechanism for the phytochemical resveratrol. One possible way to improve the system's imaging capabilities is to include coumarin-6, a fluorescent molecule [117]. The goal of Wang et al.'s micellar formulation was to treat EGFR-positive TNBC tumors by including quantum dots tagged with an anti-EGFR nanobody. Micelles containing the anti-cancer medicine aminoflavone might be visualized with the use of near-infrared fluorescent quantum dots. Via intravenous injection, the theranostic micelles significantly reduced tumor size in orthotopic TNBC animal models harboring EGFR+ malignancies [118]. Parhi and Sahoo targeted breast cancer cells that overexpress HER2 by modifying lipid-based nanoparticles and adding trastuzumab to them. The nanoparticles, which stood at around 72 nanometers in diameter, were made up of the anti-cancer drug rapamycin and the imaging agent quantum dots. In vitro studies using SKBR 3 breast cancer cells in both flat 2D and spherical 3D configurations showed that the drug was more effectively absorbed and the disease was better treated than when the drug was used alone or with unaltered nanoparticles [119].

Theranostic uses of albumin nanoparticles have also been investigated. A 151-nm nanoparticle formulation was recently developed using human serum albumin. The chemotherapy drug doxorubicin (DOX) and the MRI contrast agent gadolinium III were encapsulated in this formulation. Afterwards, the effects of this formulation were examined on TNBC xenografts, which were cultured on the chorioallantoic membrane of fertilized turkey eggs. According to the research, nanoparticles remained in tumor tissues for at least 15 hours. The percentage of proliferative Ki-67-positive cells in the xenografts was found to be much lower after treatment with these nanoparticles compared to the initial DOX therapy [120].

Research into theranostic compositions using different polymers for the treatment of breast cancer and its metastases has shown promising results. Starch, poly(methyl methacrylic acid), and polystyrene were the building blocks of a novel terpolymer developed by Li et al. The terpolymer was subsequently used to deliver DOX and gadolinium (a contrast agent for MRI) for the treatment of brain metastases resulting from breast cancer. This delivery was facilitated using a fluorescence imaging technique called near-infrared fluorophore HF750. When injected into the tail veins of mice with an severe combined immunodeficient model of brain metastatic breast cancer, the nanoparticles selectively targeted cancer cells and caused them to undergo apoptosis, while having no effect on healthy brain cells [121]. Nanoparticles and other drug delivery methods rely heavily on poly lactic-co-glycolic acid (PLGA), an FDA-approved polymer with several medicinal uses. Nanoplatelets containing DOX and other imaging agents including perfluoropentane for ultrasonic imaging, nanocarbon for photoacoustic imaging and photothermal therapy, and fluorescence imaging were recently produced by covering PLGA nanoparticles with platelet membranes. The nanoparticles had a photothermal impact when they were administered to rats with 4T1 breast tumors and then exposed to laser irradiation; this transformed light energy into heat. Additionally, DOX was released for medicinal reasons, and perfluoropentane was vaporized to enhance ultrasonic imaging as a consequence of the higher temperature [122]. Dong et al. successfully combined anti-HER2 antibodies with superparamagnetic iron oxide nanoparticles, perfluorooctyl bromide, gold-nanoshelled PLGA, and a formulation of hybrid nanoparticles. The name given to these nanoparticles was HER2-GPH nanoparticles. The accumulation of these particles was monitored using US and MRI; the HER2+ breast cancer cells were targeted by photothermal agents attached to by the particular antibody. These particles efficiently caused cell death when exposed to near-infrared light [123].

Additionally, nanotheranostics treating breast cancer using metal nanoparticles has been studied. Ruthenium (Ru) agents are a promising alternative to platinum-based chemicals for cancer therapy because of their strong anti-cancer effects with little side effects on normal cells [124]. A powerful tool in theranostic applications, Ru-based chemicals may attach to DNA via non-covalent interactions, making them imaging agents [125]. A Ru-polypyridine combination was used to create a liposome-based theranostic formulation, as reported by Shen et al. The liposome carrier made it easier for cancer cells to absorb Ru. The nanocarriers were given intravenously to rodents with an orthotopic breast cancer model based on the MDA-MB-231 human tumor type. A high concentration of the particles inside the tumor was seen 2 hours after injection, leading to a significant decrease in the growth of the TNBC tumor [126]. Our theranostic nanoformulations have the ability to target cancer cells that express the epidermal growth factor receptor. They can then deliver a Ru chemical for treatment together with a radionuclide for imaging [127]. Similarly, this formulation is helpful in treating TNBCs, which have a tendency to overexpress EGFR.

The tumor microenvironment is made up of cancer cells and various other components, including fibroblasts and immune cells. These elements can significantly influence the ability of nanoparticles to disperse effectively within the tumor. Methods that allow nanoparticles to penetrate the tumor microenvironment are highly desirable. To diagnose and particularly detect HER2+ breast cancer, a new theranostic tool created by Zeng et al. is a gold-shelled nanoparticle of HER2-DOX superparamagnetic iron oxide. When these particles were administered intravenously to BT474 breast cancer nude mice, MRI scans taken 2 hours later revealed that their concentration in the tumors had peaked. Furthermore, by lowering the number of cancer-associated fibroblasts, the use of gold shells to generate photothermal effects restructured the tumor microenvironment. Because of this, DOX was able to become more successful in its battle against tumors [128]. Because long-circulating nanoparticles generate an improvement in permeability and a retention effect, it is common for nanoparticles to collect in tumor tissues. However, in order to maintain these properties, the particle sizes are critical. Though they are very effective in penetrating tumors, small nanoparticles are easily reabsorbed into the bloodstream as they circulate. The capacity of large particles to penetrate tumor lesions is restricted, although they are easily retained in these areas [129]. Hyaluronidase can degrade the hyaluronic acid that Liu et al. developed, and CD44 allows it to target tumors selectively. For tumor fluorescence imaging, this hyaluronic acid was stabilized with cationic bovine serum albumin and included gold nanoclusters infused with indocyanine green. It also included the chemotherapeutic drug paclitaxel. The presence of hyaluronidase allowed the nanoparticles to shrink in size upon subcutaneous delivery in Balb/c mice with tumors. As a consequence, the nanoparticles were evenly distributed around the tumor [130,131].

Inducing hyperthermia in cells using nanotheranostic formulations improves cell membrane permeabilization, which, in turn, leads to tumor mass elimination. Improved cell membrane permeability and quick cell death were outcomes of the photothermal therapy carried out by Burke et al., who stimulated multiwalled carbon nanotubes using near-infrared light. When coupled with anti-cancer medications, this approach has potential for theranostic uses. Theranostics also has other promising methods, such as injectable thermoresponsive hydrogels for targeted breast cancer therapy. Wu et al. demonstrated a strong ability to generate heat when exposed to light by injecting polydopamine-coated gold nanoparticles and incorporating polyoxygen into a supramolecular thermoresponsive hydrogel known as poly(N-acryloyl glycinamide-co-acrylamide). Also, the anticancer medicine is released gradually and under supervision [132].

According to the aforementioned research, breast cancer nanotheranostics is a relatively new area of study with great promise for the future of breast cancer therapy by combining many cutting-edge technologies into a unified framework for more effective, efficient, and precisely targeted care. An anti-cancer medicinal agent and contrast chemicals for various imaging modalities may be combined to generate a single formulation for targeted theranostic medication delivery. This method offers personalized treatment and has the potential to lessen patients' pain.

## Conclusions

The integration of radiology, particularly advanced imaging techniques such as radiomics, DBT, US, and MRI, has significantly enhanced the diagnosis and treatment of breast cancer. These technologies, coupled with deep learning and AI, have shown promise in improving the accuracy of detecting malignant lesions and differentiating them from benign ones. The use of AI-driven tools, including CNNs and radiomics, can potentially reduce the workload of radiologists and improve diagnostic precision. Moreover, the development of bimodal CAD systems, which combine mammography and US, offers a higher accuracy in early detection and can minimize unnecessary biopsies. In addition, novel approaches such as nanotheranostics present a promising frontier for targeted breast cancer therapy, combining diagnostic and therapeutic modalities into a unified framework. However, despite these advancements, further research is required to validate the efficacy of these technologies in clinical settings, particularly in screening environments, and to ensure their generalizability across diverse populations.
